# SHORT-TERM MUSIC TRAINING ENHANCES INHIBITION IN AGING

**DOI:** 10.1093/geroni/igad104.1204

**Published:** 2023-12-21

**Authors:** Jennifer Bugos

**Affiliations:** University of South Florida, Tampa, Florida, United States

## Abstract

Music training was shown to benefit inhibition in adults; however, little is known about the benefits of specific types of music training and the dosages necessary to demonstrate gains in older adults. Based on the ADAM model, professional musicians use predictive temporal extrapolation to anticipate and perform coordinated bimanual movements. We applied this model to beginning adult musicians by isolating the effects of bimanual coordination when compared with non-motor standardized music interventions. We evaluated the effects of two standardized music programs (piano training, music listening instruction) on inhibition in novice adults after four-week intervals. Older adults (N=18) were recruited and randomly assigned to a manualized piano program (Keys to Staying Sharp) or music listening instruction (Music Listening Today, standard protocol). Participants completed a battery of standardized measures including measures of inhibition (Flanker, Cued Color Word Stroop) at pretesting and at each of the three follow-up intervals: 4 weeks, 8 weeks, and 12 weeks. Results of a Repeated Measures Group X Time ANOVA for the first four months of training showed reduced error rates, F(1, 14)=6.48, p=.023, and faster reaction times, F(1, 14) =5.27, p=.038 for piano training as compared to music listening instruction. Increased differences were prominent after eight-weeks of training for the piano group as compared to music listening. Data suggests that music training interventions while manualized are not created equal. Benefits from complex music performance are evident after short-term manualized music interventions. Short-term bimanual music interventions may increase cognitive performance beyond music listening instruction due to cognitive load.

